# Microscopic fabric evolution and macroscopic deformation response of gangue solid waste filler considering block shape under different confining pressures

**DOI:** 10.1038/s41598-022-11311-8

**Published:** 2022-05-05

**Authors:** Liang Chen, Junmeng Li, Dongsheng Zhang, Gangwei Fan, Wei Zhang, Yachao Guo

**Affiliations:** 1 State Key Laboratory of Coal Resources and Safe Mining, China University of Mining and Technology, Xuzhou, China; 2 School of Mines, China University of Mining and Technology, Xuzhou, China; 3 State Key Laboratory for Geomechanics and Deep Underground Engineering, China University of Mining and Technology, Xuzhou, 221116 China

**Keywords:** Environmental sciences, Natural hazards

## Abstract

The irregular shape of gangue blocks will affect the coordination structure between blocks in the crushed gangue accumulation body, and then affect the engineering mechanical properties of crushed gangue in the process of load-bearing compression. In this paper, through CT scanning experiment, particle flow numerical simulation experiment, and comprehensive application of image processing, 3D reconstruction, FLAC/PFC^3D^ continuum—discrete coupling technology, the gangue digital 3D model and the numerical model of crushed gangue particle flow under triaxial compression condition considering the real shape of the block were obtained. The microscopic fabric evolution law and macroscopic deformation response characteristics of crushed gangue considering triaxial compression condition and different confining pressures were studied. The results show that: (1) the bearing capacity of crushed gangue materials increases with the increase of confining pressure; (2) the block aggregate in the gangue sample is gradually compacted, and the lateral deformation of the sample is changed from “extruding to the axis” to “bulging to the periphery”; (3) the vertical movement of the block decreases gradually from the top to the bottom of the sample, and there is a “triangle area” of block displacement at the top and bottom of the sample; the larger the confining pressure, the smaller the vertical displacement range at the top of the sample; (4) the process of “instability and failure—optimization and reconstruction” of skeleton force chain structure occurs constantly; as confining pressure increases, the stability of skeleton force chain structure and the bearing capacity of crushed gangue sample increases; (5) under the same strain state, the greater the confining pressure, the higher the fragmentation degree of the sample. This study reveals the internal mechanism of macro deformation of crushed gangue under the triaxial compression from the perspective of the mesoscopic fabric evolution. The research results are of great significance for the selection of crushed gangue in engineering application. In addition, the research results also have a significant impact on promoting the reasonable disposal and resource utilization of gangue solid waste and protecting the ecological environment of mining areas.

## Introduction

In the process of coal mining, the discharge of crushed gangue accounts for 15–20% of coal output. At present, the crushed gangue accumulated on the ground of mining areas in China has exceeded 6 billion tons, becoming the largest industrial solid waste in China^1–3^. The accumulation of crushed gangue on the ground causes a series of environmental damage problems, posing a threat to the ecological environment and human health in mining areas, which typically include: land resource occupation, soil pollution, damage to surface vegetation, surface water and groundwater pollution, etc.^4–11^.

Hence, how to dispose and utilize the crushed gangue reasonably has become the key problem urgently needed to be solved. The main chemical composition of gangue is SiO_2_, Al_2_O_3_ etc., Containing alkali, alkaline earth metal, sulfide, organic matter etc., the density is 2.12 × 10^3^ kg m^−3^. Therefore, coal gangue is a kind of hard rock with high density and strength, which is suitable for backfill material. The gangue used in solid filling coal mining mainly comes from two sources. One source is the rock material that produced during the excavation process of rock roadway and coal roadway; the gangue from this source is called “excavation gangue,” which mainly consists of sandstone. The other source is the rock material that discharged from the coal washing process. The gangue from this source is called “washing gangue”. In recent years, with the rapid development of capital construction projects, a large number of rockfill materials are needed as fillers in many engineering fields such as rockfill dam, embankment, roadbed, foundation cushion, and filling mining. However, due to the shortage of rockfill materials, crushed gangue has been widely used as a substitute material for rockfill materials. This not only solves the problem of shortage of rockfill materials, but also realizes the large-scale treatment and resource utilization of crushed gangue^12–17^. In the application of the above engineering fields, crushed gangue serves as the main load-bearing structure of various structures (constructions), and its load-bearing compression deformation characteristics play a decisive role in the firmness and stability of various structures (constructions)^18–20^. The internal mesoscopic fabric evolution of crushed gangue during its bearing process (particle breakage, particle movement and force chain evolution) are the root causes affecting the macro bearing behavior of crushed gangue^21–23^.

At present, many scholars have studied the macroscopic mechanical behaviors of crushed gangue, such as breaking expansion characteristics, stress–strain characteristics, compaction characteristics and seepage characteristics, etc.^24–28^. Meanwhile, the influence of material ratio, particle size grading, loading conditions and other factors on its macroscopic mechanical behaviors have been widely discussed by using laboratory testing and numerical simulation methods^29–33^. Nevertheless, the research on the macro mechanical behavior of crushed gangue caused by the internal mesoscopic fabric evolution has been rarely studied, and the real shape of gangue block has not been considered in the previous studies. Therefore, there are three main problems need to be solved: (1) how does the internal mesoscopic fabric of crushed gangue evolve during the loading and compression? (2) What is the relationship between macro deformation and mesoscopic fabric evolution? (3) What is the internal mechanism leading to the macro deformation behavior of crushed gangue, such as nonlinearity, anisotropy and dilatancy?

To solve above problems, a digital 3D model of the real shape of gangue block was first established by CT scanning experiment, image processing and 3D reconstruction. Then, based on the real shape of the gangue block, a continuous–discrete coupled numerical model of crushed gangue by using the FLAC/PFC software under the triaxial compression was developed. Finally, the relationship between macro deformation and mesoscopic fabric was obtained, and the internal mechanism of macro deformation of crushed gangue was explained from the perspective of mesoscopic fabric evolution. The research results are of great significance for understanding the mechanical properties of crushed gangue materials, contributing to the selection of crushed gangue materials in engineering applications.

## Results

### Deviatoric stress–strain curves and overall deformation of crushed gangue under triaxial compression

**Figure 1:**
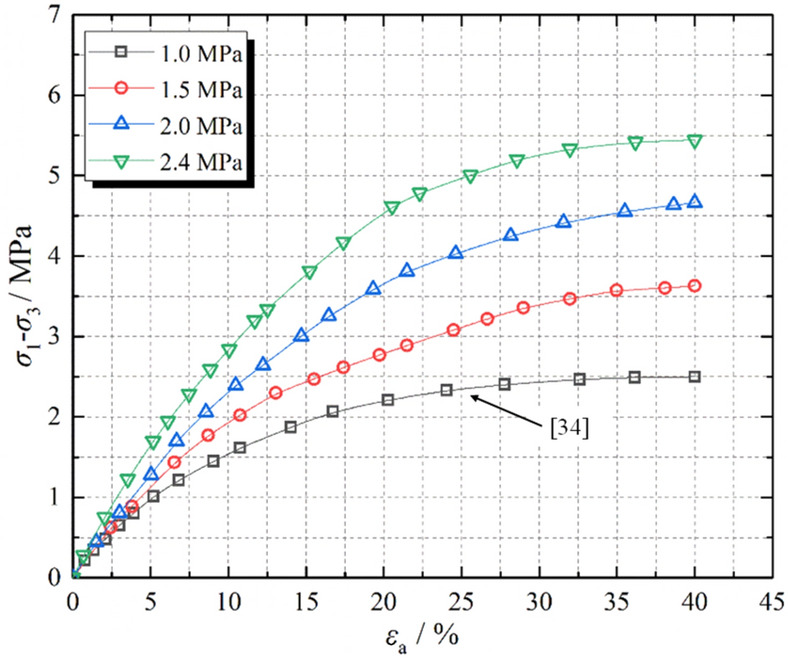
Deviatoric stress–strain curves of crushed gangue samples under different confining pressures.

**Figure 2:**
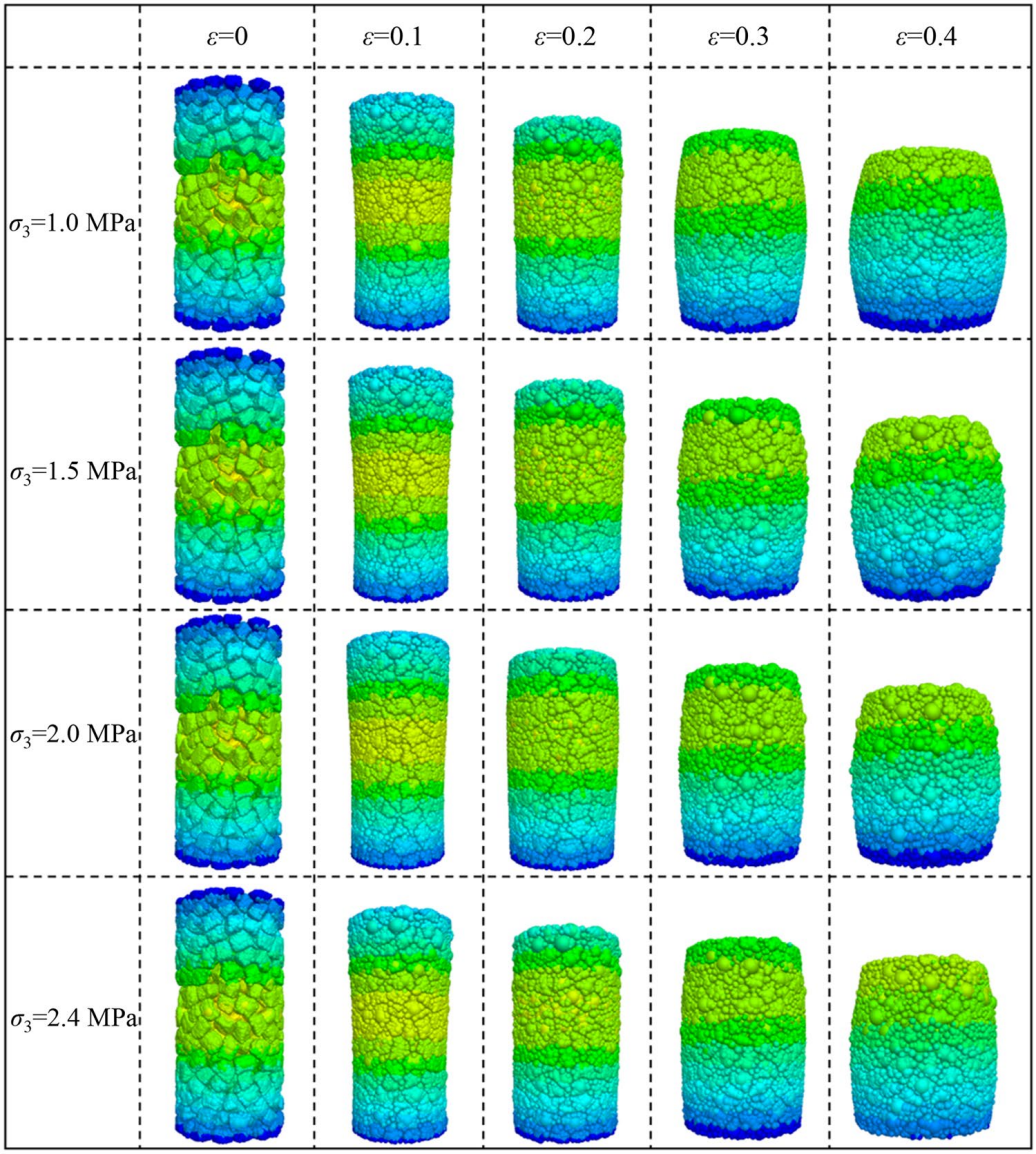
Overall deformation of crushed gangue under various triaxial compression.

The deviatoric stress–strain curve and overall deformation of crushed gangue under different confining pressures are shown in Figs. 1 and 2, respectively. It can be concluded from the Figs. 1 and 2 that in the triaxial compression process of crushed gangue, with the increase of axial deformation, its deviatoric stress gradually increases as the increasing rate decreases. The simulation results under confining pressure of 1.0 MPa are consistent with those in literature^34^ (the black curve in Fig. 1). Hence, the accuracy of the numerical simulation model is reliable. Under the same strain condition, the deviatoric stress of crushed gangue increases as the confining pressure increases. When ε = 0.4, the maximum axial stress σ_1_ of crushed gangue is 3.50 MPa, 4.63 MPa, 5.66 MPa and 6.44 MPa under confining pressures of 1.0 MPa, 1.5 MPa, 2.0 MPa and 2.4 MPa, respectively, indicating that the bearing capacity of crushed gangue increases as the confining pressure increases. In addition, the bulging deformation is mostly obvious at the low confining pressure. With the increase of confining pressure, the degree of bulging deformation gradually decreases. The reasons are as follows: the larger the confining pressure, the higher the restraint degree of the block in the sample, the more difficult the lateral deformation of the block. As a result, the bulging deformation of the sample is less significant.

### Vertical movement evolution law of block of crushed gangue under triaxial compression

**Figure 3:**
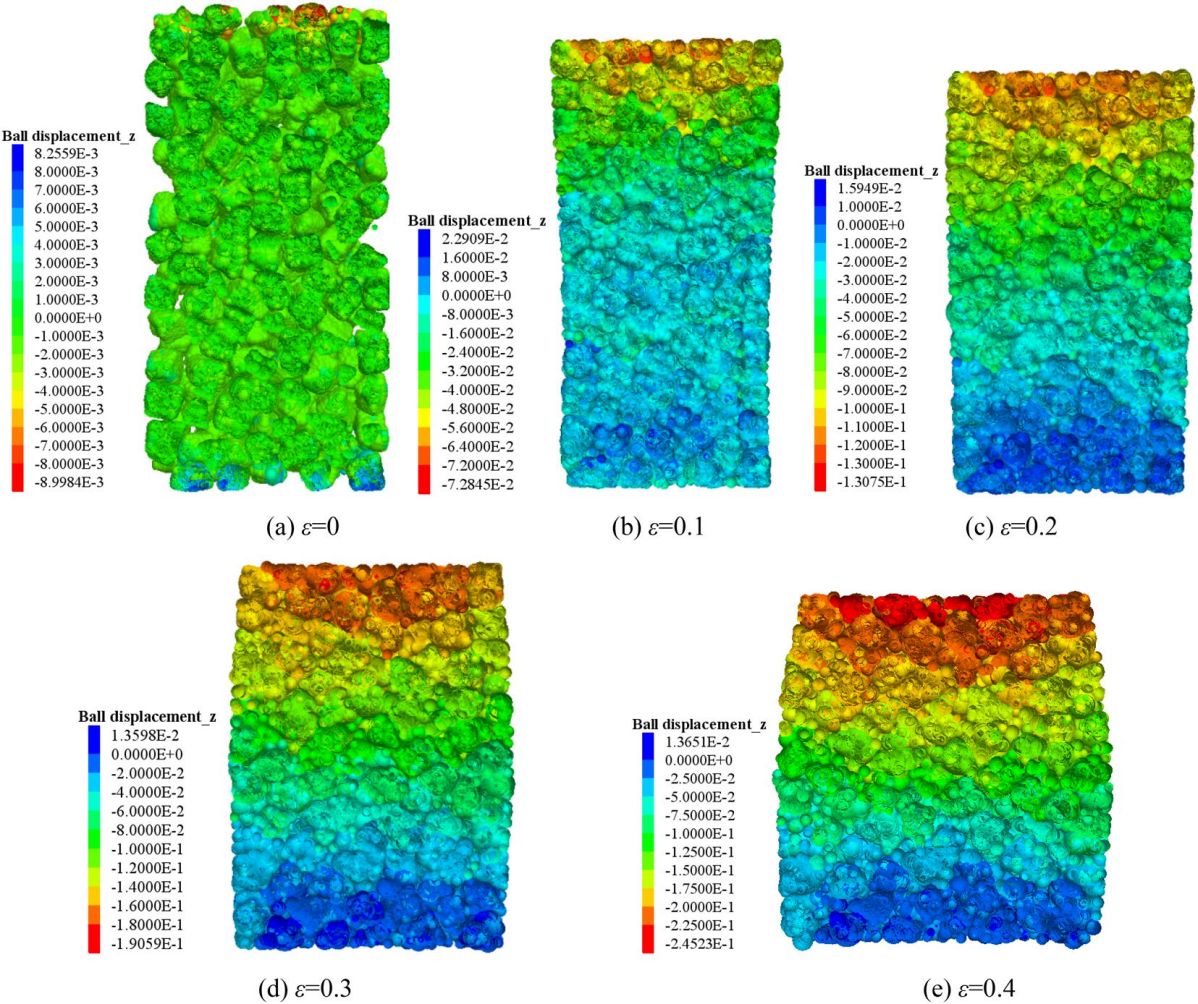
Vertical movement characteristics of the block at different strain states (σ_3_ = 1.0 MPa).

**Figure 4:**
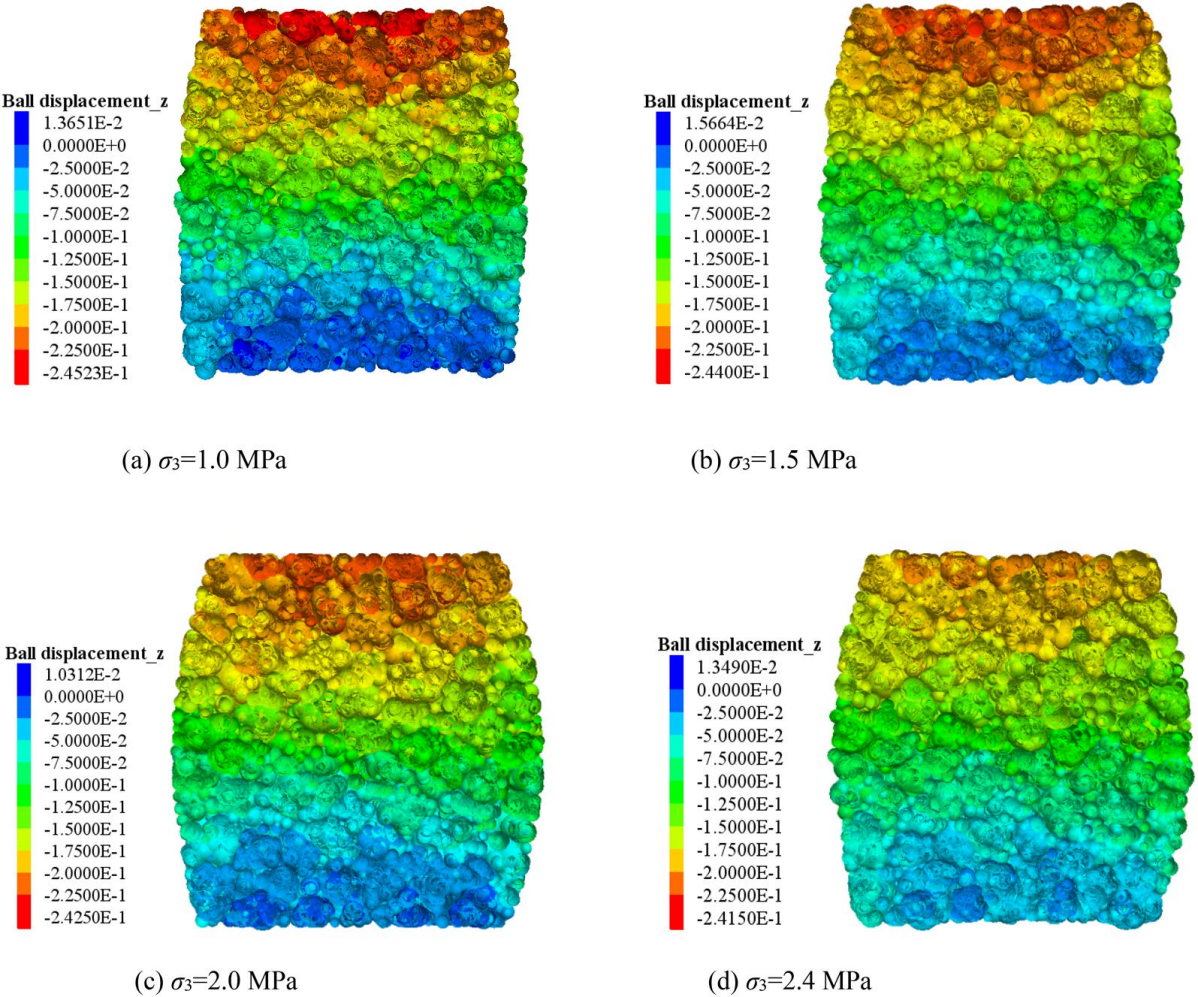
Vertical movement characteristics of the block under different confining pressures (*ε* = 0.4).

The vertical movement of the block in the crushed gangue are obtained considering different strain states at σ_3_ = 0.1 MPa, as shown in the cloud map of Fig. 3. The vertical movements of the block considering different confining pressures at ε = 0.4 are shown in Fig. 4. It can be concluded from Figs. 3 and 4 that

1. The vertical movement amount of the block in the crushed gangue decreases from the top to the bottom of the gangue sample in the process of triaxial compression. This is because the block at the top of the gangue sample is first disturbed by the loading plate, and the displacement is the largest. With the increase of the distance between the block and the loading plate, the disturbance degree gradually decreases and the displacement decreases gradually.
2. The vertical movement of gangue block increases gradually with the increasing compression deformation. However, due to the constraint of the rigid loading plate and rigid base, the “triangle area” of the block displacement appears at the top and bottom of the sample, and the “triangle area” becomes more and more obvious with the loading process. The vertical displacement of the block in the top “triangle area” is significantly greater than other blocks in the same layer; the vertical displacement of the block in the bottom “triangle area” is small, which is obviously lower than other blocks. The above results show that: the vertical displacement of blocks in the same layer is different in the process of triaxial compression. In other words, when the vertical displacement of block occurs in the sample, the adjacent blocks have obvious dislocation.
3. the triangular area of vertical displacement at the top of the crushed gangue sample decreases as the confining pressure increases. The reasons are as follows: the greater the confining pressure, the higher the restraint degree of the block in the sample, the more difficult the lateral displacement of the block, and the less significant the dislocation between adjacent blocks in the same cross section. Finally, the “triangle area” of vertical displacement at the specimen top is less obvious.

### Lateral movement evolution law of block of crushed gangue under triaxial compression

**Figure 5:**
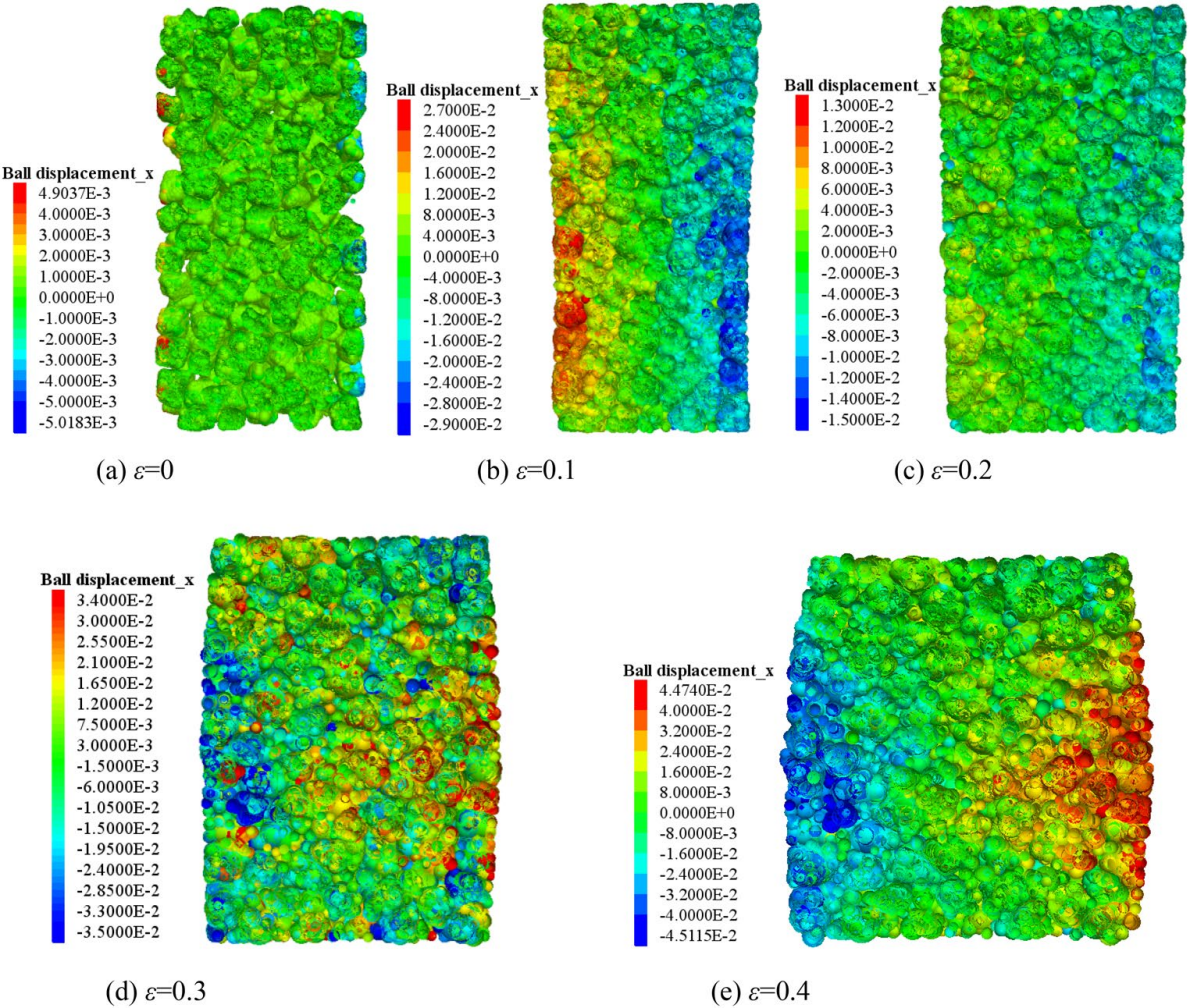
Lateral movement characteristics of the block at different axial strains (*σ*_3_ = 1.0 MPa).

**Figure 6:**
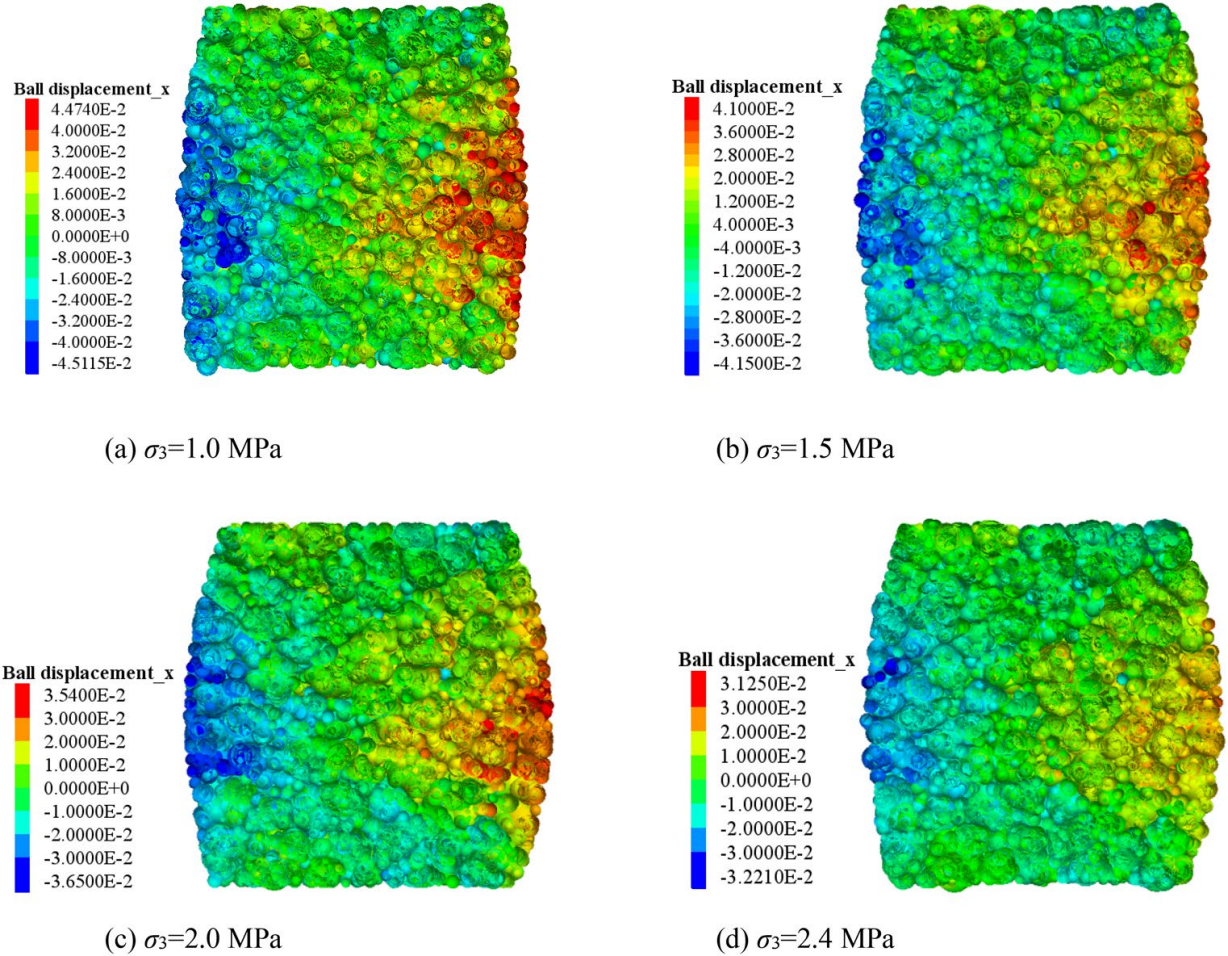
Lateral movement characteristics of the block under various confining pressures (ε = 0.4).

The lateral movement of the blocks in the crushed gangue are obtained considering various strain states at σ_3_ = 1.0 MPa, as shown in the cloud map of Fig. 5. The lateral movement of the blocks are obtained considering different confining pressures at ε = 0.4, as shown in Fig. 6. It can be concluded from Figs. 5 and 6 that

1. The lateral movement of the gangue block changes from gathering towards the axis to expanding outward in the process of triaxial compression, when the axial deformation is increased. In the initial stage of loading, due to the loose sample, under the action of confining pressure, the block in the sample move laterally in the axial direction. With the loading process, the lateral movement of the block is difficult to be restrained by the confining pressure. Under the action of axial load, the block begins to move outward. Moreover, since the sample generally has the end effect in the triaxial loading process, the closer the position is to the two ends of the sample, the smaller the lateral displacement of the block is. Then a “triangle area” of lateral displacement inside the block is formed both sides of the sample. This indicates that the lateral displacement of the block in the middle of the sample is the largest, and the lateral displacement of the block decreases gradually in the direction of the two sections of the sample. With the axial deformation increasing, the “triangle area” becomes more and more obvious. The above results reveal the deformation evolution mechanism of the crushed gangue samples from “extruding to the axis” to “bulging to the periphery” during the loading and compression from the perspective of mesoscopic fabric evolution of block movement.
2. When the specimen is compressed to the same strain, with the increase of the confining pressure, the “triangle area” of lateral displacement of the crushed gangue sample becomes smaller. This is mainly caused by the limitation of confining pressure on the block's lateral displacement. The greater confining pressure leads to higher degree of restraint on the block in the sample and the greater difficulty of block lateral displacement.

### Skeleton force chain evolution law of the specimen under triaxial compression

**Figure 7:**
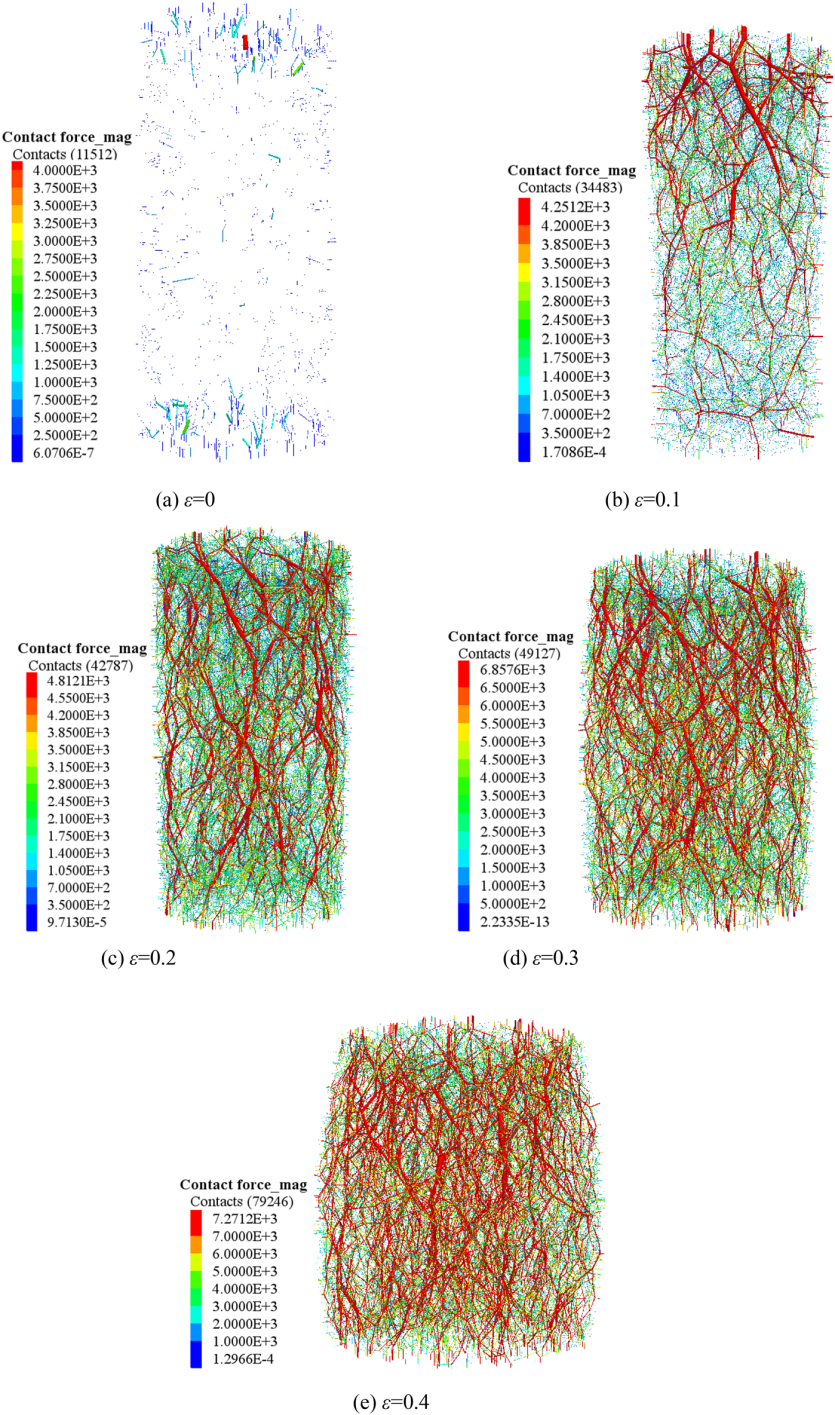
Distribution characteristics of the skeleton force chain at different axial strain state (*σ*_3_ = 1.0 MPa).

**Figure 8:**
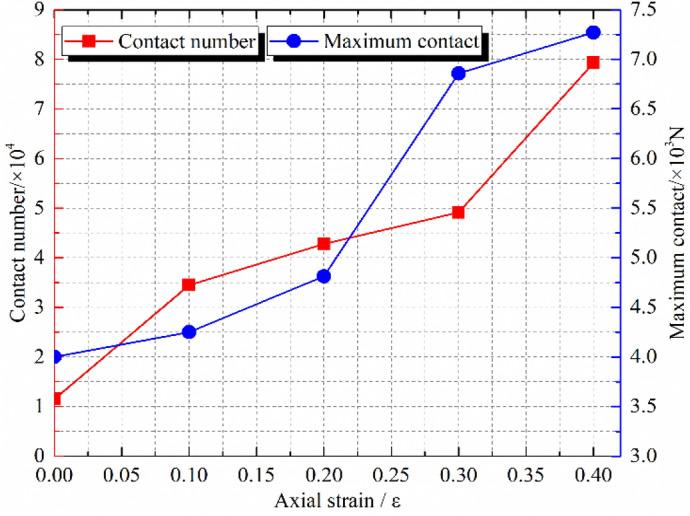
Contact number and maximum contact force vary with axial strain (*σ*_3_ = 1.0 MPa).

**Figure 9:**
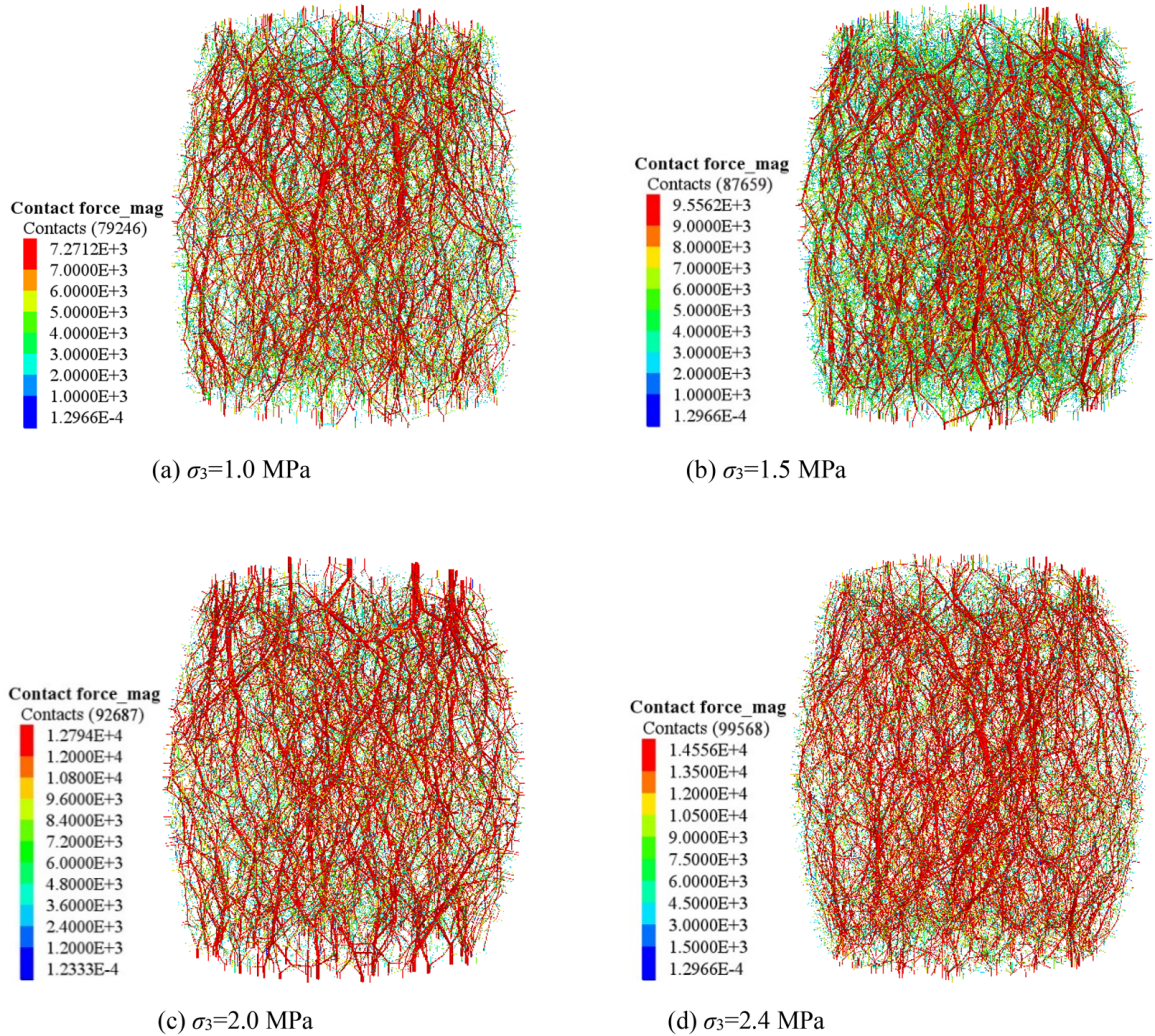
Distribution characteristics of the skeleton force chain under various confining pressures (*ε* = 0.4).

**Figure 10:**
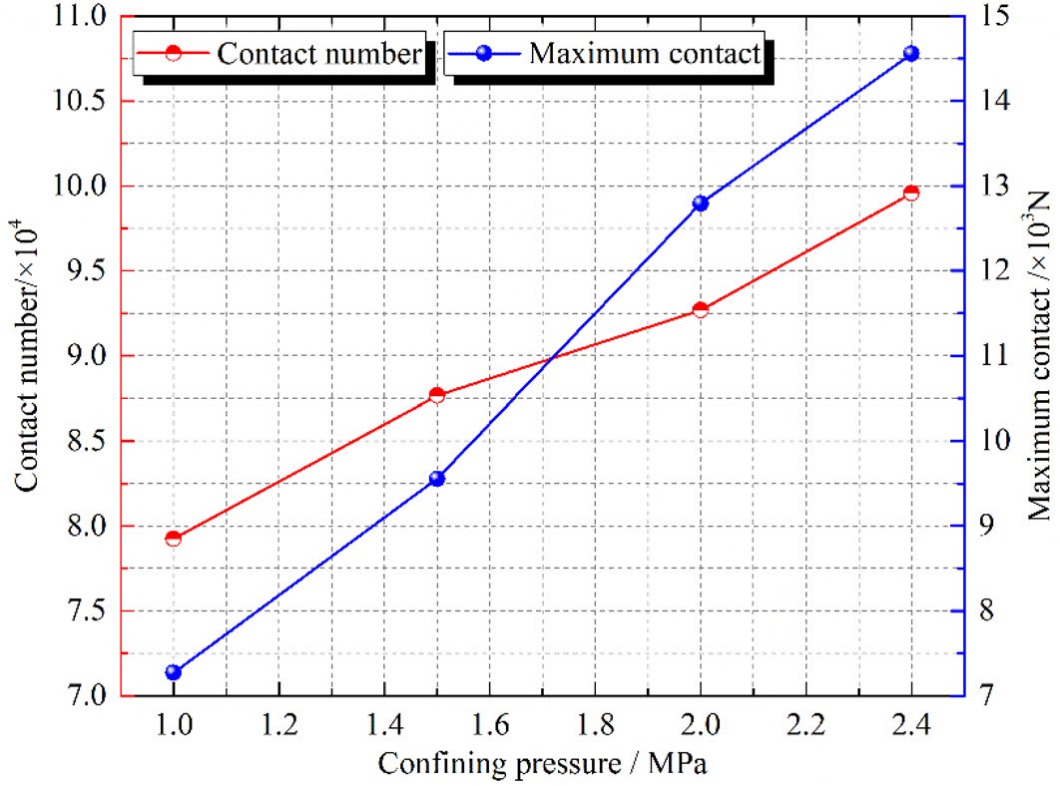
Contact number and maximum contact force vary with confining pressures (ε = 0.4).

PFC^3D^ alternates force–displacement laws and Newton's laws of motion throughout the calculation cycle. The contact force of the contact part is updated by force–displacement law. In the triaxial compression process, most of the external load is mainly borne by the skeleton force chain. Under the condition of confining pressure of 1.0 MPa, the sample composed of crushed gangue are compressed to different strain states (ε = 0.0, 0.1, 0.2, 0.3 and 0.4), then the skeleton force chain distribution characteristics in the crushed gangue samples are obtained, as shown in Fig. 7. Furthermore, the relations of contact number-axial strain and maximum contact force-axial strain in the samples are also discussed in Fig. 8. Under the confining pressures of σ_3_ = 1.0 MPa, 1.5 MPa, 2.0 MPa and 2.4 MPa, the crushed gangue samples are compressed to ε = 0.4, and the evolution characteristics of skeleton force chain distribution are obtained, as shown in Fig. 9. The relations of contact number-confining pressure and maximum contact force-confining pressure in the samples are also discussed in Fig. 10. The red chains in the Figs. 7 and 9 represent the skeleton force chain, other colors reflect general contact forces, and the thickness of the skeleton force chain shows the magnitude of the contact force. It can be seen from Figs. 7, 8, 9 and 10 that.

1. As the skeleton force chain structure gradually forms, the process of “failure—reconstruction” begins to occur continuously. However, the stability of the skeleton force chain structure becomes stronger with the increasing axial strain. This is because the most of the external load is borne by the skeleton force chain structure. With the increase of the external force, the block in the load-bearing framework is first crushed due to the stress concentration, leading to the instability and failure of the bearing skeleton structure. Then, a series of phenomena, such as displacement, rotation of the block in the sample, and filling of the small block into the gap between the large blocks are caused. As a result, the stability of the large block is improved, the internal structure of the sample is adjusted and optimized, and a more stable skeleton force chain structure is formed. Therefore, the bearing capacity and deformation resistance of the sample are improved.
2. Confining pressure affects the stability of skeleton chain structure of the samples during triaxial compression. As the confining pressure increases, the stability of the skeleton force chain structure gradually improved, the total contact number and maximum contact force between particles also gradually increase. The reasons are as follows: the greater the confining pressure, the higher the degree of restraint on the block in the sample, the greater the difficulty of block movement during the compression deformation, the more stable the block in the skeleton force chain structure. This will result in a more stable and stronger skeletal force chain structure which greatly improves the bearing capacity of crushed gangue sample. When the crushed gangue samples are loaded to ε = 0.4 under the conditions of σ_3_ = 1.0 MPa, 1.5 MPa, 2.0 MPa and 2.4 MPa, the total number of contacts in the samples are 79,246, 87,659, 92,687 and 99,568, respectively, with an increase of 25.6%. The maximum contact forces in the samples are 7271 N, 9556 N, 12,794 N and 14,556 N, respectively. It can be deduced that the increasing confining pressure can significantly enhance the bearing capacity of crushed gangue.

### Block fragmentation evolution law of the specimen under triaxial compression

**Figure 11:**
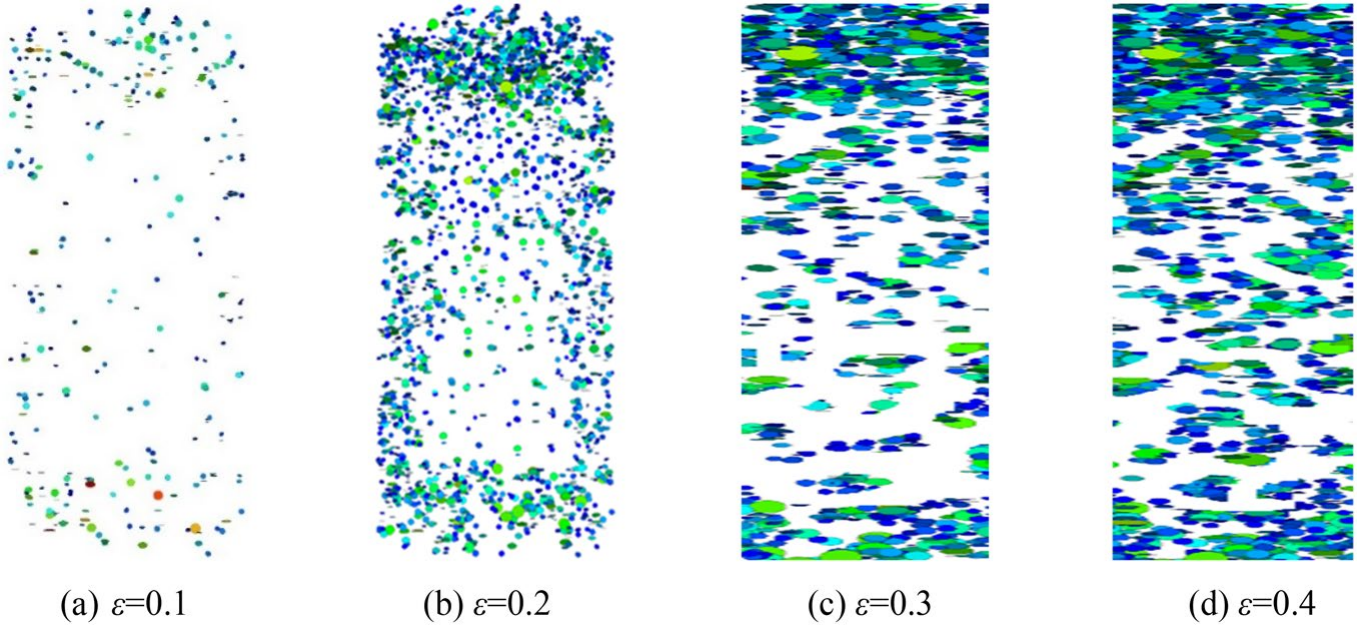
Temporal and spatial distribution characteristics of block fragmentation at various axial strains (*σ*_3_ = 1.0 MPa).

**Figure 12:**
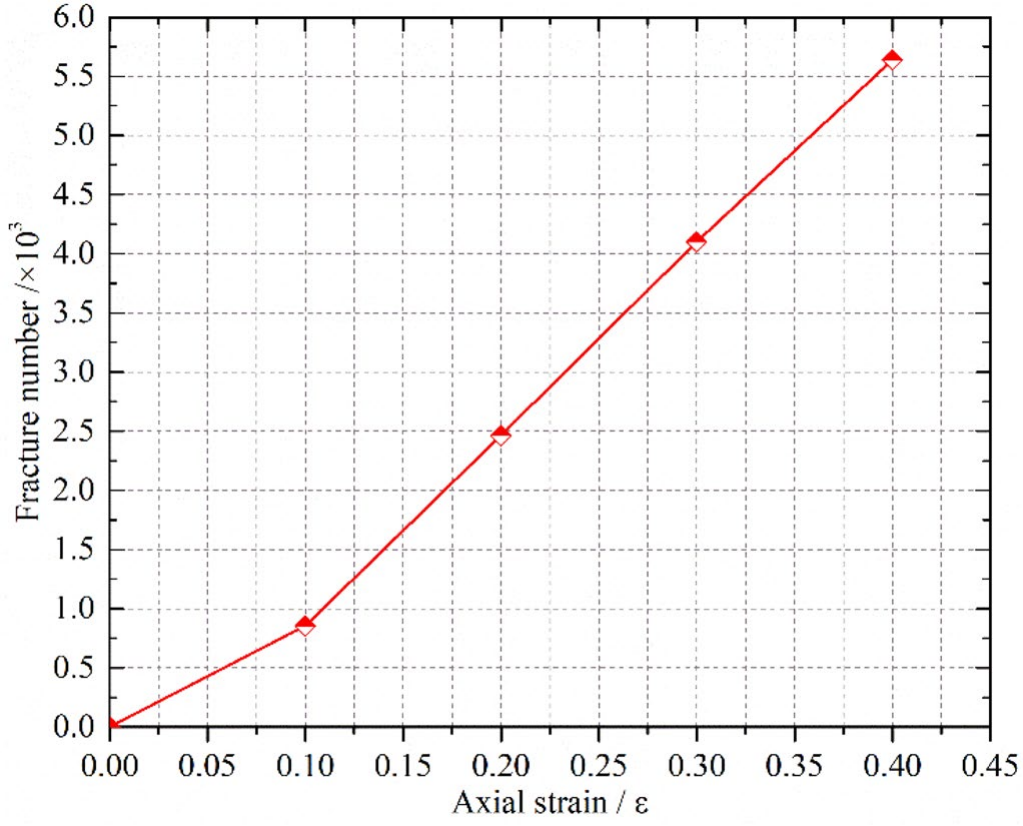
The number of crushed blocks vary with axial strain (*σ*_3_ = 1.0 MPa).

**Figure 13:**
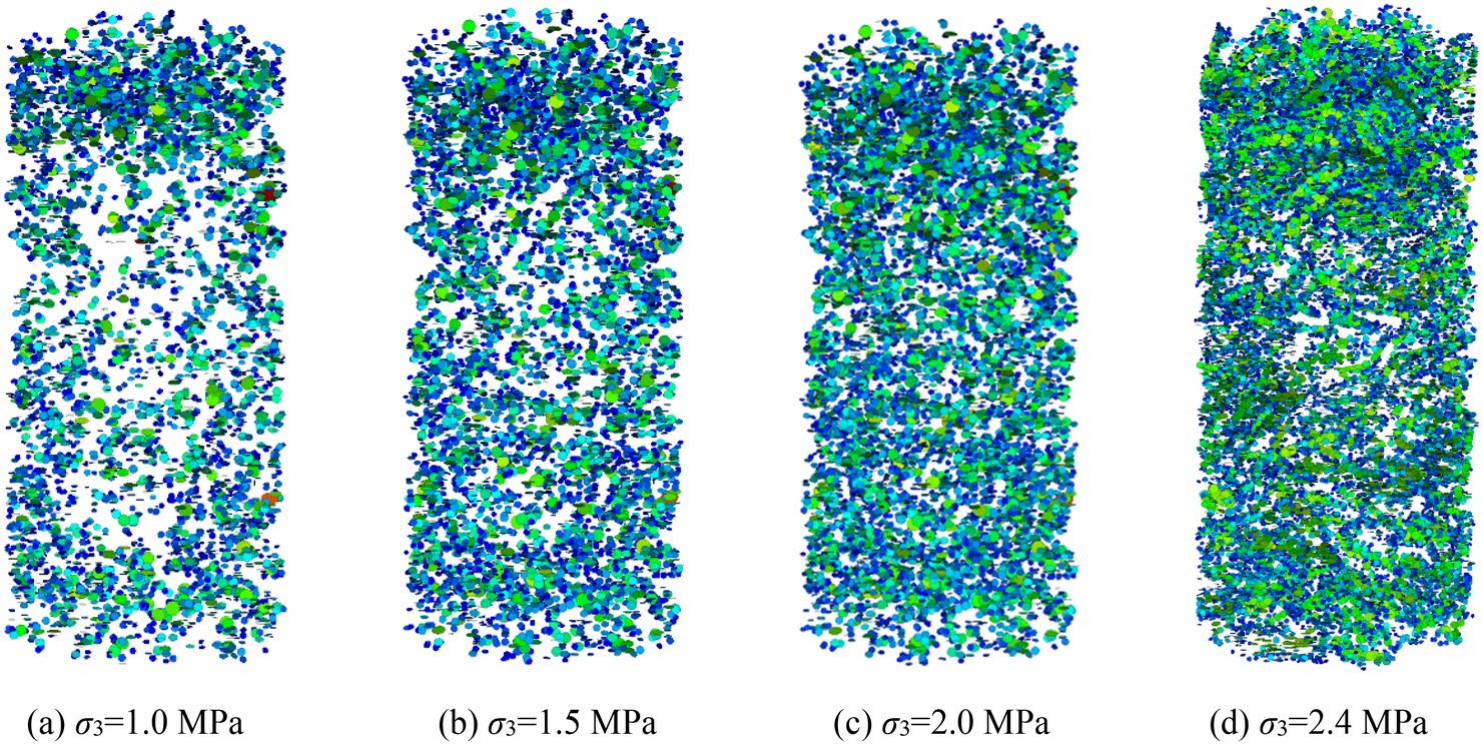
Spatial distribution characteristics of block fragmentation under various confining pressures (*ε* = 0.4).

**Figure 14:**
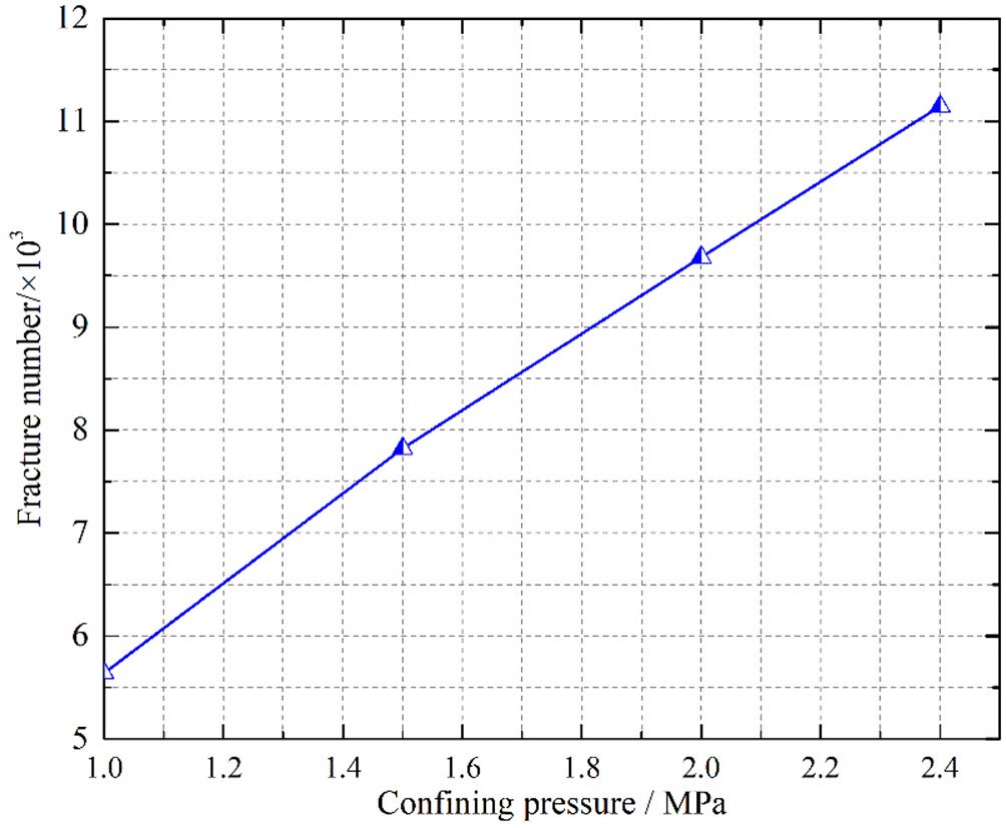
Number statistics of the crushed blocks under various confining pressures (*ε* = 0.4).

The fish function was written to traverse and judge the state of parallel bond in the numerical model during the calculation process. When the parallel bond is broken, it is considered that the block has broken damage, and it is recorded as a fragmentation, and the spatial coordinates of fragmentation are recorded. When the confining pressure is σ_3_ = 1.0 MPa, crushed gangue samples is compressed to ε = 0.1, 0.2, 0.3 and 0.4, and then the spatial–temporal distribution characteristics of blocks are obtained, as shown in Fig. 11. The crushed block number are also counted in the Fig. 12. Under the confining pressure of σ_3_ = 1.0 MPa, 1.5 MPa, 2.0 MPa and 2.4 MPa, the crushed gangue sample is compressed to ε = 0.4, and the spatial distribution characteristics of blocks are obtained, as shown in Fig. 13. The test results are shown in Fig. 14. It can be seen from Figs. 11, 12, 13 and 14 that.

1. under the triaxial compression condition, in the loading process, the block fragmentation in the sample is gradually developed, and the amount of fragmentation increases gradually. Besides, the following spatial distribution characteristics are presented: the fragmentation amount of the block gradually decreases (except for the bottom) from the top to bottom and shows an obvious distribution characteristic of top > bottom > middle. This is due to the end effect at the top and bottom of the sample, and the strength of the rigid loading plate and rigid base is much higher than that of the gangue block. When the top and bottom blocks of the sample contact with the rigid loading plate and the rigid base, the gangue blocks are more likely to be broken. At the same time, the external load is gradually transmitted from the top to the bottom, and most of the load is transferred through the skeleton force chain structure. The block in the skeleton force chain bears the higher load and is more likely to be crushed.
2. Confining pressure has a significant influence on the fragmentation degree of gangue samples. The higher the confining pressure, the higher the fragmentation degree. When the crushed gangue samples are loaded to ε = 0.4 for σ_3_ = 1.0 MPa, 1.5 MPa, 2.0 MPa and 2.4 MPa, the number of crushed gangue samples is 5639, 7822, 9678 and 11,146, respectively, with an increase of 97.7%. This is because the higher the confining pressure, the higher the restraint degree of the block, and the stronger the stability of the skeleton force chain structure formed in the sample. When the specimen is loaded to the same strain state, the greater the pressure, the higher the indirect contact force of the block, and the more likely the block to be crushed.

## Discussion and conclusion

Many scholars have investigated the compressive deformation behavior of crushed gangue by laboratory test and numerical modelling, of which the results indicated that the block shape and confining pressure have an extremely important influence on the macroscopic and microscopic deformation response characteristics of crushed gangue^22,35^. Liu et al. proposed a macadam random generating model by using computational geometry algorithms, and then a generating method code was written in MATLAB software and embedded in the Particle Flow PFFC^2^^D^ to investigate the implemented several numerical biaxial tests under different confining pressures^35^. Dongtao Wu et al. investigated the bearing characteristic, deformation and failure rules of red-sandstone gangue of different sizes from macroscopic and microscopic perspectives by physical compression testing and numerical simulation of grain flow PFC2D^36^. However, the real shape of crushed gangue was ignored in the above studies. It is not inconsistence with the practice engineering. Junmeng li et al. obtained the real shape of four irregular gangue (Knife shape, bar shape, disk shape and cube shape) and investigated the influence of real shape on the overall appearance deformation, vertical compression displacement, lateral movement, meso-fabric and skeleton force chain of evolution characteristic of the blocks in crushed gangue samples. But, the gradual evolution laws of above parameters under different confining pressures were not considered^34^. In this paper, based on the previous research method by Junmeng Li^34^, the numerical model of the FLAC/PFC^3D^ continuous–discrete coupled particle flow of the crushed gangue considering the effect of real shape under various confining pressure is established. The mesoscopic fabric evolution law and compression deformation characteristics of specimens under different confining pressures are studied, and the internal mechanism of macro deformation of crushed gangue is revealed from the perspective of mesoscopic fabric evolution. The main conclusions are as follows:

1. The bearing capacity of crushed gangue gradually increases with the increasing confining pressure. The maximum axial stress σ_1_ inside the sample under the confining pressure of 1.0 MPa, 1.5 MPa, 2.0 MPa and 2.4 MPa and ε = 0.4 is 3.50 MPa, 4.63 MPa, 5.66 MPa and 6.44 MPa, respectively. In the process of triaxial compression, the block aggregate of crushed gangue is gradually compacted. The lateral displacement characteristic of the crushed gangue gradually changes from “extrusion to axis” to “bulging to the periphery” as the axial deformation increases, and then the shape of the specimen is gradually “drum-shaped”.
2. The vertical movement amount of the block inside the sample decreases gradually from the top to the bottom in the process of triaxial compression, and then the “triangle area” of the block displacement appears at the top and bottom of the sample. The vertical displacement of the block in the top “triangle area” is significantly greater than other blocks, while the vertical displacement of the block in the bottom “triangle area” is the smallest, indicating that the vertical displacement of the block in the sample is accompanied by obvious dislocation between adjacent blocks. Meanwhile, the larger the confining pressure, the smaller the “triangle area” of the vertical displacement on the top of the crushed gangue sample.
3. The lateral movement of the block in the sample changes from gathering towards the axis to expanding outward, and the “triangle area” of lateral displacement is gradually formed on both sides of the sample in the process of triaxial compression of crushed gangue. As the axial deformation increases, the “triangle area” of lateral displacement becomes more and more obvious. The larger the confining pressure, the smaller the “triangle area” range of lateral displacement.
4. During the loading process of crushed gangue, the blocks of large particle size contact with each other and gradually form a skeleton force chain structure, of which the structure extends from the specimen top to the bottom, and finally runs through the whole sample. With the loading going on, the skeleton force chain structure continuously undergoes the process of “instability and failure—optimization and reconstruction”. However, the stability and the bearing capacity of the samples are gradually enhanced. With the increase of confining pressure, the stability of the skeleton force chain structure is much stronger, the total number and maximum contact force between particles are also larger, and the bearing capacity of crushed gangue is stronger.
5. With the loading progress, the block fragmentation in the specimen is gradually developed, and the block fragmentation at the top of the specimen is mostly obvious, followed by the bottom. From the top to the bottom of the sample, the fragmentation amount of the block decreases gradually (except for the bottom). Under the same compression conditions, the higher the confining pressure, the higher the degree of fragmentation.
6. According to the research results of this paper, in the solid backfill coal mining engineering practice, in order to ensure the best bearing capacity of backfill materials and achieve better backfill effect, after the broken gangue is filled into the goaf, the tamping force of the tamping mechanism of the hydraulic support of solid filling mining should be set above 2 MPa to give sufficient confining pressure to the filling body.

## Material and methods

### Research ideas

**Figure 15:**
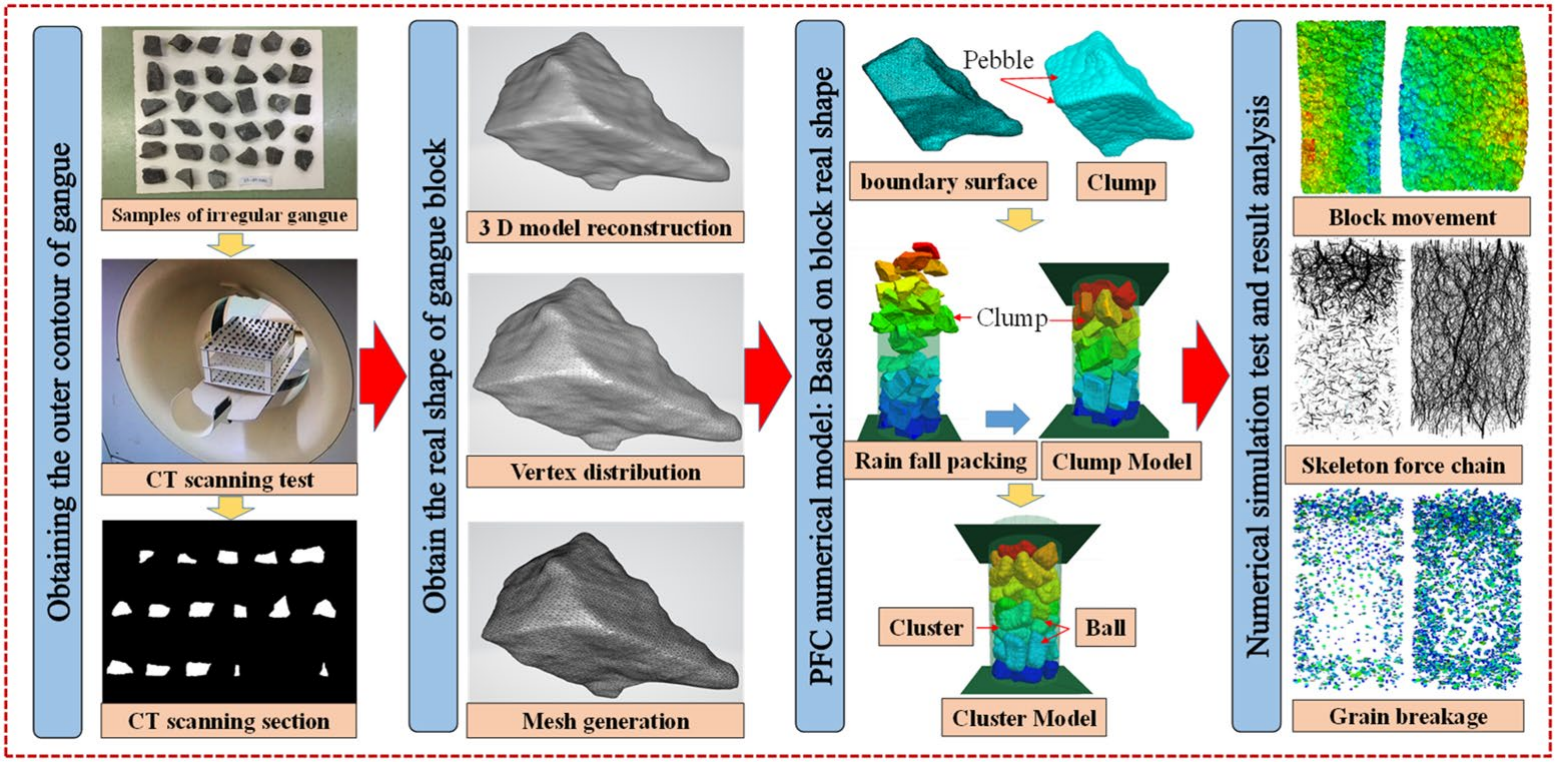
Research design on mesoscopic fabric evolution and macro deformation response characteristics of crushed gangue under the triaxial compression^34^.

The whole research process mainly includes (1) the acquisition of the real shape of gangue; (2) the development of a continuous–discrete coupled particle flow numerical model of crushed gangue under the triaxial compression based on the real shape of blocks, (3) the numerical simulation test of particle flow in crushed gangue under the triaxial compression, and (4) the analysis and discussion of the test results. Figure 15 shows the specific research ideas of this study. It should be noted that the research process is similar with the reference^34^ and obtained by modified from the previous publication. However, as mentioned in the discussion, the research content has a larger difference.

### Random acquisition of the real shape of gangue blocks

Firstly, a series of CT slices of the external contour of the irregular gangue block was obtained through CT scanning test. Secondly, a series of processing was carried out on the CT slices by MIMICS software. The processing process mainly includes superposition, cutting, binarization, 3D reconstruction calculation, smoothing processing and mesh optimization. Finally, the real shape digital 3D reconstruction model of irregular gangue block is obtained, as shown in Fig. 15.

### Development of particle flow model of crushed gangue under the triaxial compression

**Figure 16:**
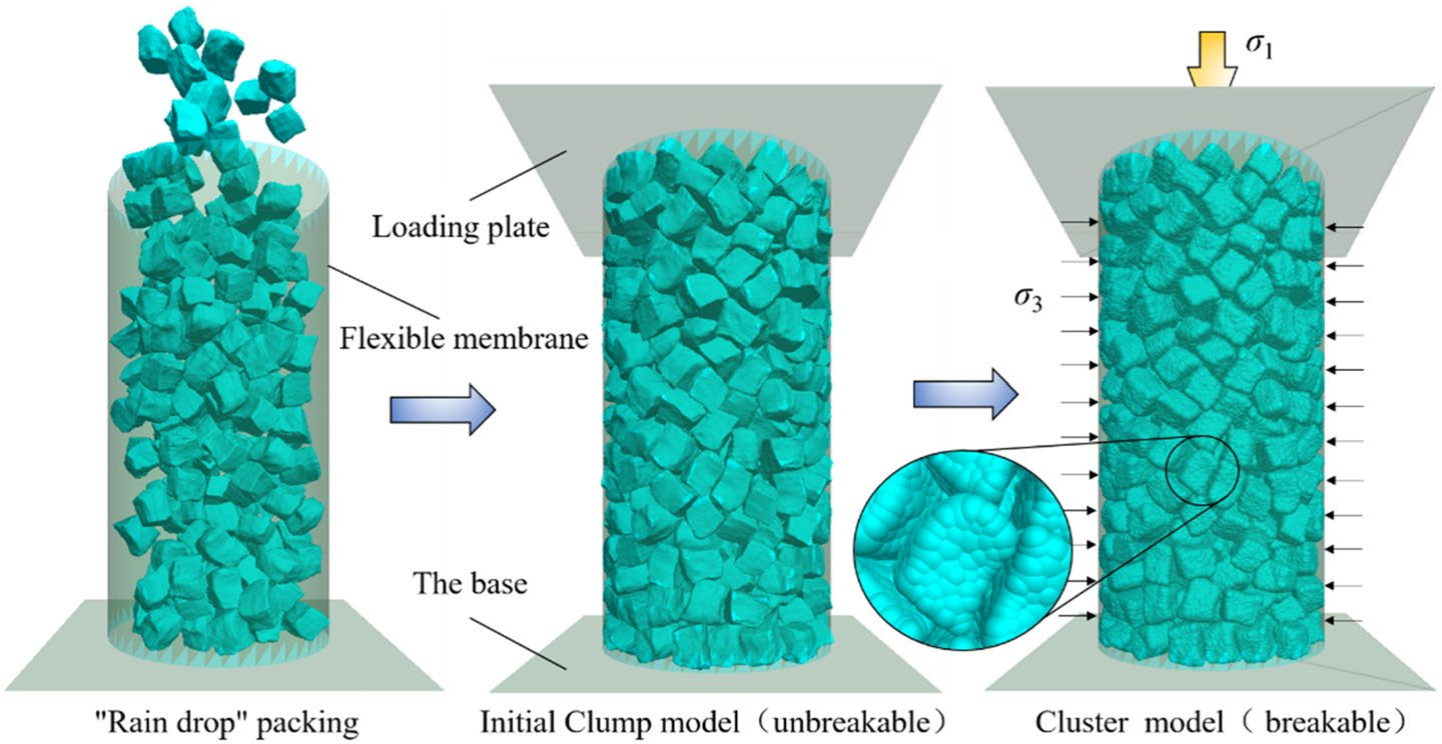
Numerical calculation model of continuous–discrete coupled particle flow of crushed gangue under triaxial compression based on the real shape of the block.

The surface of the gangue block is angular and concave–convex, which leads to the significant occlusive effect and stress concentration between blocks during the loading and compression. The occlusive effect between blocks restricts its slip and rotation, and the stress concentration promotes the fragmentation of gangue. As a result, the compression deformation characteristics of crushed gangue are significantly affected. In this paper, by using the method in literature^34^ and through the secondary development of the program, the boundary surface program of the digital 3D model of gangue blocks with real shape was written, which was imported into the PFC^3D^ numerical simulation software, and a Φ300 mm × H600 mm FLAC/PFC continuous–discrete coupled particle flow numerical 3D model of cylindrical crushed gangue under triaxial compression was established. Parameter calibration is an important step of the numerical model involved in this paper to ensure the reliability of numerical simulation results. Before modeling, a large-scale tri-axial compression test was designed under the same conditions, and the deviated stress–strain curves of graded gangue with the same particle size were obtained under the same compression conditions. Then the parameters of the numerical model were calibrated repeatedly until the deviated stress–strain curve calculated by the numerical model was basically consistent with the laboratory results. Then based on this numerical model, the internal fabric evolution characteristics of broken gangue during loading and compression process are studied. This is shown in Fig. 16.

### Particle flow numerical simulation of crushed gangue under the triaxial compression

The gangue used in this paper is sandstone excavation gangue from Chensilou Coal Mine. In this paper, crushed gangue samples with a single grading (45–50 mm) under different confining pressures were used to study the macro deformation response characteristics (including the stress–strain, overall deformation) and mesoscopic fabric evolution characteristics (including the block movement, skeleton force chain distribution evolution, block fragmentation). In solid backfill coal mining, the initial confining pressure of filling materials is provided by the tamping mechanism of solid backfill coal mining hydraulic support, and its tamping force is generally designed to be 1.0–3.0 MPa. Therefore, the laboratory confining pressure is designed as 1.0 MPa, 1.5 MPa, 2.0 MPa and 2.4 MPa respectively in this test. Table 1 shows the specific test scheme design.

**Table 1:** Particle flow numerical simulation of the crushed gangue under the triaxial compression.

Test scheme no.	Particle size	Confining pressure (MPa)	Research contents
#1	45–50 mm	1.0	Stress–strain, overall deformation, block movement, skeleton force chain distribution evolution, block fragmentation
#2		1.5	
#3		2.0	
#4		2.4	

## References

[1] Li M, Zhang J, Gao R (2016). Compression characteristics of solid wastes as backfill materials. Adv. Mater. Sci. Eng..

[2] Li B, Yan H, Zhang J, Zhou N (2020). Compaction property prediction of mixed gangue backfill materials using hybrid intelligence models: A new approach. Constr. Build. Mater..

[3] Cheng Y, Hongqiang M, Hongyu C, Jiaxin W, Jing S, Zonghui L, Mingkai Y (2018). Preparation and characterization of coal gangue geopolymers. Constr. Build. Mater..

[4] Wu D, Yang B, Liu Y (2015). Transportability and pressure drop of fresh cemented coal gangue-fly ash backfill (CGFB) slurry in pipe loop. Powder Technol..

[5] Kabala C, Galka B, Jezierski P (2020). Assessment and monitoring of soil and plant contamination with trace elements around Europe’s largest copper ore tailings impoundment. Sci. Total Environ..

[6] Li M, Zhang J, Song W, Germain DM (2019). Recycling of crushed waste rock as backfilling material in coal mine: Effects of particle size on compaction behaviours. Environ. Sci. Pollut. Res..

[7] Bian Z, Miao X, Lei S, Chen S-E, Wang W, Struthers S (2012). The challenges of reusing mining and mineral-processing wastes. Science.

[8] Zhou B, Shao M, Wen M, Wang Q, Horton R (2010). Effects of coal gangue content on water movement and solute transport in a China Loess Plateau Soil. Clean: Soil, Air, Water.

[9] Neto HFDS, Pereira WVDS, Yan ND, Souza ESD, Fernandes AR (2020). Environmental and human health risks of arsenic in gold mining areas in the eastern Amazon. Environ. Pollut..

[10] Li J, Yan X, Cao Z, Yang Z, Liang J, Ma T, Liu Q (2020). Identification of successional trajectory over 30 years and evaluation of reclamation effect in coal waste dumps of surface coal mine. J. Clean. Prod..

[11] Li J, Wang J (2019). Comprehensive utilization and environmental risks of coal gangue: A review. J. Clean. Prod..

[12] Long G, Li L, Li W, Ma K, Dong W, Bai C, Zhou JL (2019). Enhanced mechanical properties and durability of coal gangue reinforced cement-soil mixture for foundation treatments. J. Clean. Prod..

[13] Li L, Long G, Bai C, Ma K, Wang M, Zhang S (2020). Utilization of coal gangue aggregate for railway roadbed construction in practice. Sustainability.

[14] Liu Z, Zhang C, Qu X (2020). Study on the parameter optimization and strength mechanism of coal gangue emulsified asphalt mixture. Adv. Mater. Sci. Eng..

[15] Ma H, Zhu H, Wu C, Chen H, Sun J, Liu J (2020). Study on compressive strength and durability of alkali-activated coal gangue-slag concrete and its mechanism. Powder Technol..

[16] Sahu SN, Sharma K, Barma SD, Pradhan P, Nayak BK, Biswal SK (2019). Utilization of low-grade BHQ iron ore by reduction roasting followed by magnetic separation for the production of magnetite-based pellet feed. Metallurg. Res. Technol..

[17] Zhang J, Zhang Q, Huang Y, Liu J, Zhou N, Zan D (2011). Strata movement controlling effect of waste and fly ash backfillings in fully mechanized coal mining with backfilling face. Min. Sci. Technol. (China).

[18] Huang Y, Li J, Ma D, Gao H, Guo Y, Ouyang S (2019). Triaxial compression behaviour of gangue solid wastes under effects of particle size and confining pressure. Sci. Total Environ..

[19] Feng G, Du X, Zhang Y (2019). “Optical-acoustic-stress” responses in failure progress of cemented gangue-fly ash backfill material under uniaxial compression. Nondestruct. Test. Eval..

[20] Li J, Huang Y, Chen Z, Zhang J, Jiang H, Zhang Y (2019). Characterizations of macroscopic deformation and particle crushing of crushed gangue particle material under cyclic loading: In solid backfilling coal mining. Powder Technol..

[21] Cheng L, Qin Y, Li X, Zhao X (2020). A laboratory and numerical simulation study on compression characteristics of coal gangue particles with optimal size distribution based on shape statistics. Math. Probl. Eng..

[22] Li M, Li A, Zhang J, Huang Y, Li J (2019). Effects of particle sizes on compressive deformation and particle breakage of gangue used for coal mine goaf backfill. Powder Technol..

[23] Huang Y, Li J, Song T, Sun Q, Kong G, Wang F (2017). Microstructure of coal gangue and precipitation of heavy metal elements. J. Spectrosc..

[24] Li M, Zhang J, Li A, Zhou N (2020). Reutilisation of coal gangue and fly ash as underground backfill materials for surface subsidence control. J. Clean. Prod..

[25] Zhou M, Dou Y, Zhang Y, Zhang Y, Zhang B (2019). Effects of the variety and content of coal gangue coarse aggregate on the mechanical properties of concrete. Constr. Build. Mater..

[26] Du X, Feng G, Qi T, Guo Y, Zhang Y, Wang Z (2019). Failure characteristics of large unconfined cemented gangue backfill structure in partial backfill mining. Constr. Build. Mater..

[27] Zhang J, Li M, Liu Z, Zhou N (2017). Fractal characteristics of crushed particles of coal gangue under compaction. Powder Technol..

[28] Zhang J, Liu Y, Zhou N, Li M (2018). Pore pressure evolution and mass loss of broken gangue during the seepage. R. Soc. Open Sci..

[29] Ma D, Duan H, Liu J, Li X, Zhou Z (2019). The role of gangue on the mitigation of mining-induced hazards and environmental pollution: An experimental investigation. Sci. Total Environ..

[30] Zhang Y, Zhou W, Li M, Chen Z (2018). Experimental study on compression deformation and permeability characteristics of grading broken gangue under stress. Processes.

[31] Zhou N, Han X, Zhang J, Li M (2016). Compressive deformation and energy dissipation of crushed coal gangue. Powder Technol..

[32] Huang Y, Li J, Teng Y, Dong X, Wang X, Kong G, Song T (2017). Numerical simulation study on macroscopic mechanical behaviors and micro-motion characteristics of gangues under triaxial compression. Powder Technol..

[33] Gong P, Ma Z, Zhang RR, Ni X, Liu F, Huang Z (2017). Surrounding rock deformation mechanism and control technology for gob-side entry retaining with fully mechanized gangue backfilling mining: A case study. Shock. Vib..

[34] Li J, Huang Y, Pu H, Gao H, Li Y, Ouyang S, Guo Y (2021). Influence of block shape on macroscopic deformation response and meso-fabric evolution of crushed gangue under the triaxial compression. Powder Technol..

[35] Liu Z, Zhou N, Zhang J (2013). Random gravel model and particle flow based numerical biaxial test of solid backfill materials. Int. J. Min. Sci. Technol..

[36] Wu D, Luo F, Li M, Diao Y, Guo Y, Xu P (2021). Macroscopic and microscopic study on the compression bearing characteristics and deformation failure mechanism of gangue with different particle sizes. Powder Technol..

